# Sirtuin 2 Deficiency Increases Bacterial Phagocytosis by Macrophages and Protects from Chronic Staphylococcal Infection

**DOI:** 10.3389/fimmu.2017.01037

**Published:** 2017-08-28

**Authors:** Eleonora Ciarlo, Tytti Heinonen, Charlotte Théroude, Jacobus Herderschee, Matteo Mombelli, Jérôme Lugrin, Marc Pfefferlé, Beatrice Tyrrell, Sarah Lensch, Hans Acha-Orbea, Didier Le Roy, Johan Auwerx, Thierry Roger

**Affiliations:** ^1^Infectious Diseases Service, Department of Medicine, Lausanne University Hospital, Epalinges, Switzerland; ^2^Department of Biochemistry, University of Lausanne, Epalinges, Switzerland; ^3^Laboratory for Integrative and Systems Physiology, Ecole Polytechnique Fédérale de Lausanne, Lausanne, Switzerland

**Keywords:** sirtuin, innate immunity, cytokine, macrophage, phagocytosis, sepsis, histone deacetylase, metabolism

## Abstract

Sirtuin 2 (SIRT2) is one of the seven members of the family of NAD^+^-dependent histone deacetylases. Sirtuins target histones and non-histone proteins according to their subcellular localization, influencing various biological processes. SIRT2 resides mainly in the cytoplasm and regulates cytoskeleton dynamics, cell cycle, and metabolic pathways. As such, SIRT2 has been implicated in the pathogenesis of neurodegenerative, metabolic, oncologic, and chronic inflammatory disorders. This motivated the development of SIRT2-directed therapies for clinical purposes. However, the impact of SIRT2 on antimicrobial host defense is largely unknown. Here, we address this question using SIRT2 knockout mice. We show that SIRT2 is the most highly expressed sirtuin in myeloid cells, especially macrophages. SIRT2 deficiency does not affect immune cell development and marginally impacts on intracellular signaling and cytokine production by splenocytes and macrophages. However, SIRT2 deficiency enhances bacterial phagocytosis by macrophages. In line with these observations, in preclinical models, SIRT2 deficiency increases survival of mice with chronic staphylococcal infection, while having no effect on the course of toxic shock syndrome toxin-1, LPS or TNF-induced shock, fulminant *Escherichia coli* peritonitis, sub-lethal *Klebsiella pneumoniae* pneumonia, and chronic candidiasis. Altogether, these data support the safety profile of SIRT2 inhibitors under clinical development in terms of susceptibility to infections.

## Introduction

Innate immune cells are at the vanguard of host defense against microbial infections. Monocytes/macrophages and dendritic cells (DCs) sense microbial or danger-associated molecular patterns (MAMPs and DAMPs released by injured or stressed cells) through pattern recognition receptors (PRRs) such as toll-like receptors (TLRs), NOD-like receptors, C-type lectins, scavenger receptors, RIG-I-like receptors, and intra-cytosolic DNA sensors (1, 2). The interaction of MAMPs or DAMPs with PRRs triggers the mitogen-activated protein kinases (MAPKs), nuclear factor-κB (NF-κB), and interferon (IFN) response factor signaling pathways that coordinate immune gene expression. The cellular and soluble mediators mobilized upon infection tightly regulate the development of the inflammatory response, the establishment of antimicrobial cellular and humoral responses, and the restoration of homeostasis once pathogens have been contained or eradicated.

The superfamily of histone deacetylases (HDACs) comprises eleven Zn-dependent HDACs (HDAC1-11) and seven NAD^+^-dependent sirtuins (SIRT1-7). HDACs are epigenetic erasers catalyzing histone deacetylation, chromatin compaction, and transcriptional repression. In addition, HDACs target thousands of non-histone proteins affecting many biological processes (3). The subfamily of sirtuins attracted much interest when sirtuins were proposed to promote longevity and represent attractive therapeutic targets for age-related pathologies, such as type 2 diabetes, as well as neurodegenerative, cardiovascular, and oncologic diseases (4–6). Our knowledge about the impact of sirtuins on innate immune responses is limited. Most studies have focused on SIRT1 and SIRT6, resulting in an overall complex picture attributing both proinflammatory and anti-inflammatory properties to sirtuins (7).

Sirtuin 2 (SIRT2) was originally reported to colocalize with the microtubule network and to deacetylate α-tubulin (8). Indeed, SIRT2 is mainly cytoplasmic, although it can translocate during the G2/M transition phase of cell cycle into the nucleus where it deacetylates histone H4 lysine 16 (9). Of note, SIRT2 possesses a proficient demyristoylation activity, the physiological relevance of which remains to be established (10). By targeting numerous proteins besides histones, SIRT2 regulates cytoskeleton dynamics, cell cycle, and metabolic pathways including inhibition of adipogenesis and promotion of lipolysis and gluconeogenesis. Additionally, SIRT2 can act as a tumor suppressor gene, and is involved in myelogenesis and other brain functions. Accordingly, SIRT2 has been implicated in tumorigenesis and neurodegeneration, and likely plays a role in metabolic disorders, such as obesity and type 2 diabetes (11–13).

With respect to inflammatory processes, SIRT2 was first shown to deacetylate NF-κB p65, resulting in the expression of a subset of p65-dependent genes in mouse embryonic fibroblasts (14). SIRT2 deficiency sustained brain inflammation in a model of traumatic brain injury and increased the severity of collagen-induced arthritis and colitis (15–17). However, SIRT2 deficiency was also reported to decrease NF-κB p65-mediated inflammatory response, renal tubular inflammation, and ischemia reperfusion-induced hepatocellular inflammation (18–20). Finally, in an experimental stroke model, SIRT2 deficiency preserved neurological functions without affecting inflammatory parameters (21). Overall, the function of SIRT2 in sterile and chronic inflammatory disorders appears to be context dependent.

Because sirtuins are pleiotropic and in consideration of the development of sirtuin-targeting drugs for clinical conditions, we sought to delineate the role of SIRT2 in the innate immune response. To this end, we used SIRT2 knockout mice to investigate the response of immune cells to immunological and microbial stimuli using *in vitro* experiments and *in vivo* preclinical models. Preclinical models included models of shock as well as Gram-negative and Gram-positive bacterial infections and fungal infection. Overall, SIRT2 deficiency does not modulate cytokine production by innate immune cells, but enhances bacterial phagocytosis by macrophages. SIRT2 deficiency protects from chronic staphylococcal infection, while having no impact on toxic shock, endotoxemia, fulminant peritonitis, non-lethal pneumonia, and chronic candidiasis. These data largely support the safety, in terms of susceptibility to infections, of SIRT2 inhibitors developed for clinical applications.

## Materials and Methods

### Mice, Cells, and Reagents

8- to 12-week-old female BALB/cByJ mice, C57BL/6J mice (Charles River Laboratories, Saint-Germain-sur-l’Arbresle, France), and SIRT2 knockout mice backcrossed 12 times on a C57BL/6J background (15) were used. Mice were housed under specific pathogen-free conditions and free of mouse norovirus. Splenocytes were cultured in RPMI 1640 medium containing 2 mM glutamine, 50 µM 2-ME, 100 IU/ml penicillin, 100 µg/ml streptomycin (Invitrogen, San Diego, CA) and 10% heat-inactivated fetal calf serum (FCS; Sigma-Aldrich, St. Louis, MO) (22). Bone marrow (BM) cells were cultured in IMDM (Invitrogen) containing 50 µM 2-ME, penicillin, streptomycin, and 10% FCS. Medium was supplemented with 20 ng/ml M-CSF, 20 ng/ml GM-CSF plus 20 ng/ml IL-4 (ProSpec, East Brunswick, NJ) or 200 ng/ml FMS-like tyrosine kinase 3 ligand (Flt3L, Shenandoah biotechnology, Warwick, PA) to generate BM-derived macrophages (BMDMs), BM-derived dendritic cells (BMDCs), or Flt3L-DCs, respectively. BMDCs were collected after 6 days. BMDMs and Flt3L-DCs were collected after 7 days of culture. Cells (1, 5, and 20 × 10^5^) were seeded in 96-well, 24-well or 6-well plates in complete medium without growth factors and antibiotics unless specified.

*Salmonella minnesota* ultra pure lipopolysaccharide (LPS) was from List Biologicals Laboratories (Campbell, CA), Pam_3_CSK_4_ from EMC microcollections (Tübingen, Germany), CpG ODN 1826 (CpG) from InvivoGen (San Diego, CA, USA), toxic shock syndrome toxin-1 (TSST-1) and staphylococcal enterotoxin B (SEB) from Toxin Technology (Sarasota, FL, USA), concanavalin A and phytohemagglutinin (PHA) from Sigma-Aldrich, and anti-CD3ε and anti-CD28 antibodies (clones 145-2C11 and 37.51) from eBioscience (San Diego, CA, USA). Clinical strains of *Escherichia coli* (*E. coli*) O18, *E. coli* J5, *E. coli* O111, *Salmonella enterica* serovar Typhimurium C5 (*Salmonella* Typhimurium), *Klebsiella pneumoniae* caroli (*K. pneumoniae*), *Neisseria meningitis, Streptococcus pneumoniae, Staphylococcus aureus* AW7 (*S. aureus*), and Group B *Streptococcus* (GBS) were grown in brain heart infusion broth (BD Biosciences, Erembodegem, Belgium) (23–27). *Candida albicans* 5102 (*C. albicans*) (22) was cultured in yeast extract–peptone–dextrose (BD Biosciences). Microorganisms were washed in PBS and adjusted at 10^10^ CFU/ml. For *in vitro* stimulation, bacteria were heat-inactivated for 2 h at 56°C. Nocodazole and 2-deoxyglucose were from Sigma-Aldrich, cytochalasin D from Millipore (Billerica, MA, USA).

### RNA Analyses

Total RNA was isolated, reverse transcribed (RNeasy and QuantiTect reverse transcription kits, Qiagen, Hilden, Germany), and used in real-time PCRs conducted with a QuantStudio™ 12K Flex system (Life Technologies, Carlsbad, CA, USA). Reactions consisted of 1.25 µl cDNA, 1.25 µl H_2_O, 0.62 µl 10 nM primers [Table S1 in Supplementary Material and Ref. (28, 29)], and 3.12 µl Fast SYBR^®^ Green Master Mix (Life Technologies) and were tested in triplicate. Gene specific expression was normalized to hypoxanthine guanine phosphoribosyl transferase expression. Sirt2 expression levels in organs were extracted from the BioGPS resource (http://biogps.org).

### Western Blot Analyses

Nuclear and total protein extracts were submitted to PAGE and transferred onto nitrocellulose membranes (30, 31). Membranes were incubated with antibodies directed against SIRT2, acetylated α-tubulin, total α-tubulin, total and phosphorylated ERK1/2, p38, JNK, and NF-κB p65 and TATA-box binding protein (used as a control of nuclear extracts) (see antibody description in Table S2 in Supplementary Material), then with a secondary horseradish peroxidase-conjugated antibody (Sigma-Aldrich) (32). Blots were imaged with the enhanced chemiluminescence Western blotting system (GE Healthcare, Little Chalfont, Royaume-Uni). Images were recorded using a Fusion Fx system (Viber Lourmat, Collégien, France).

### Flow Cytometry

Single cell suspensions from thymus and spleen, or BMDMs were enumerated and incubated with 2.4G2 monoclonal antibody (mAb). Immune cell subpopulations were tracked by staining performed using mAbs described in Table S2 in Supplementary Material. Splenic CD4^+^ CD25^+^ Foxp3^+^ cells were detected using The Mouse Regulatory T Cell Staining Kit (eBioscience). Data were acquired using a LSR II flow cytometer (BD Biosciences) and analyzed using FlowJo Version 10.2 software (FlowJo LLC, Ashland, OR, USA) (33).

### Proliferation Assay

The proliferation of 1.5 × 10^5^ splenocytes cultured for 48 h in 96-well plates was quantified by measuring ^3^H-thymidine incorporation over 18 h (34).

### Cytokine Measurements

Cytokine concentrations were quantified using DuoSet ELISA kits (R&D Systems, Abingdon, UK) or Luminex assays (Affimetrix eBioscience, Vienna, Austria) (35).

### *In Vivo* Models

8- to 12-week-old female SIRT2^+/+^ and SIRT2^−/−^ mice (6–13 mice per group) matched for age were used. To analyze the response to TSST-1, mice were challenged intraperitoneally (i.p.) with TSST-1 (0.5 mg/kg). Models of endotoxic shock were performed by challenging mice i.p. with LPS (10 and 25 mg/kg). To induce TNF shock, mice were sensitized with d-galactosamine (30 mg/kg i.p., Sigma-Aldrich) just before being challenged with TNF (25 mg/kg i.p., Preprotech, Rocky Hill, NJ, USA). Bacterial sepsis was induced by challenging mice i.p. with 10^5^ CFU *E. coli* O18, intravenously (i.v.) with 10^7^ CFU *S. aureus* or 10^5^ CFU *C. albicans* or intranasally (i.n.) with 30 CFU *K. pneumoniae*. Blood and spleen were collected 0, 1, 6, 8, 24, or 48 h post-challenge to quantify cytokines and bacteria (28). Body weight loss, severity score, and survival were registered at least once daily. The severity score was graded from 1 to 5 (36). Animals were euthanized when they met a severity score of 4. Two to three operators performed animal follow-up.

### Phagocytosis Assays

Fluoresbrite^®^ Yellow Green Microspheres (Polysciences Inc, Warrington, PA, USA) or FITC-labeled bacteria were added to cells at a ratio of 10 beads or bacteria/cell. After 1 h, cells were washed, incubated for 1 min with trypan blue (0.25 mg/ml) and analyzed by flow cytometry. When specified, beads were opsonized with serum for 30 min at 37°C. To assess phagocytosis of live bacteria, BMDMs (in quadruplicates or sextuplates) were incubated for 1 h with *E. coli* O18, *S. aureus*, and GBS (10 bacteria/cell). Non-adherent and extracellular bacteria were removed by washing and killed by a 30-min exposure to 100 µg/ml gentamicin (Essex Chemie, Luzern, Switzerland; for *E. coli* and GBS) or 10 µg/ml ciprofloxacin (Fresenius Kabi, Oberdorf, Switzerland; for *S. aureus*). Serial dilutions of cell lysates were plated on agar plates. Colonies were enumerated to calculate the number of phagocytosed bacteria.

### Glycolytic Activity

The glycolytic activity of BMDMs was analyzed using a 96-well format Seahorse XFe^®^ system and the Seahorse XF Glycolysis Stress Test Kit (Agilent Technologies, Santa Clara, CA, USA). Briefly, 4 × 10^4^ BMDMs were plated in 96-well plates in IMDM medium. The next day, cells were incubated with or without 5 × 10^7^ CFU/ml heat-killed *S. aureus* and rested 1 h in Seahorse medium without glucose. The glycolytic capacity was assessed by measuring the extracellular acidification rate following the sequentially addition of 10 mM glucose, 1 µM oligomycin, and 50 mM 2-deoxy-glucose (2-DG) according to manufacturer’s instructions.

### Statistical Analyses

Comparisons between the different groups were performed by analysis of variance followed by two-tailed unpaired Student’s *t*-test. The Kaplan–Meier method was used for building survival curves and differences were analyzed by the log-rank sum test. All analyses were performed using PRISM (GraphPad Software). *P* values were two-sided, and *P* < 0.05 was considered to indicate statistical significance.

## Results

### SIRT2 Is Highly Expressed by Myeloid Cells

**Figure 1:**
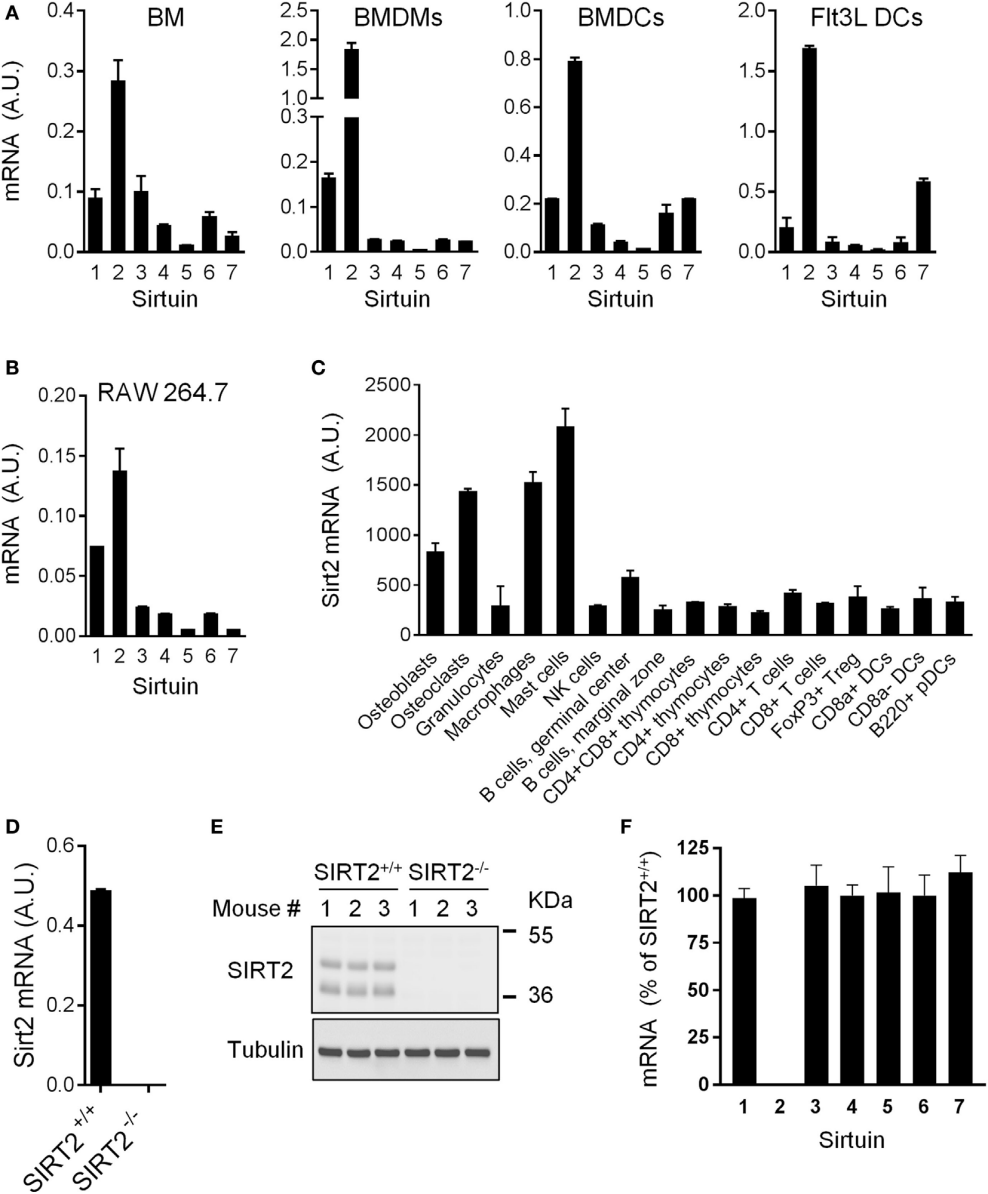
Sirt2 is strongly expressed in myeloid cells. **(A,B)** Sirt1-7 mRNA levels in bone marrow (BM), BM-derived macrophages (BMDMs) and dendritic cells (BMDCs), and Flt3L-derived DCs **(A)** and RAW 264.7 macrophages **(B)** analyzed by RT-PCR and normalized to Hprt mRNA levels. Data are means ± SD of triplicate samples from one experiment performed with four BALB/c mice **(A)** or three preparations of RAW 264.7 macrophages **(B)**. **(C)** Sirt2 mRNA expression levels in a panel of cells (extracted from http://biogps.org). **(D,E)** Sirt2 mRNA **(D)** and Sirtuin 2 (SIRT2) protein **(E)** expression in SIRT2^+/+^ and SIRT2^−/−^ BMDMs assessed by RT-PCR and western blotting, respectively. Data are means ± SD from one experiment performed with three mice **(D)**. **(F)** Sirt1-7 mRNA expression levels in SIRT2^−/−^ BMDMs, expressed relative to the mRNA levels in SIRT2^+/+^ BMDMs set at 100%. Data are means ± SD from one experiment performed with three mice. A.U., arbitrary units. Full-length blots of panel **(E)** are presented in Figure S1 in Supplementary Material.

SIRT1-7 mRNA expression was quantified in BM, BMDMs, and DCs (conventional BMDCs and Flt3L-derived DCs) (Figure 1A). SIRT2 was the most highly expressed sirtuin in all populations. SIRT2 was also the predominantly expressed sirtuin in RAW 264.7 macrophages and in the spleen, liver, and kidneys (Figure 1B and data not shown). Western blot analyses confirmed SIRT2 protein expression in BMDMs (see below). Primary osteoblasts, osteoclasts, macrophages, and mast cells expressed 2.6-fold to 7.1-fold higher levels of SIRT2 mRNA than granulocytes, NK cells, T cells, B cells, DCs, and thymocytes (Figure 1C). Overall, SIRT2 was highly expressed by myeloid-derived cells, suggesting that it could play a role in the control of immune responses. To address this question, SIRT2-deficient mice were used [Figures 1D,E and Ref. (15)].

### SIRT2 Deficiency Has No Major Impact on the Development of Immune Cells and Host Response to TSST-1

SIRT2^−/−^ mice were described previously (15). These mice were born at the expected Mendelian ratio and developed without abnormalities. SIRT2 mRNA and protein were undetectable in SIRT2^−/−^ BMDMs (Figures 1D,E). Expression levels of Sirt1 and Sirt3-7 mRNA were unaltered in SIRT2^−/−^ BMDMs (Figure 1F), suggesting that the lack of SIRT2 was not compensated by an increase in expression of other sirtuins.

Compared to SIRT2^+/+^ mice, SIRT2^−/−^ mice expressed normal proportions and absolute numbers of CD4/CD8 double negative (DN1–4), double positive, and single positive (SP) thymocytes (Table 1). Additionally, SIRT2^−/−^ mice had normal populations of splenic T cells (DN, SP, naïve, and memory), B cells (immature and mature B cells), DCs (B220^−^ CD11c^+^ cDCs and B220^+^ CD11c^+^ pDCs), and Foxp3^+^ regulatory T cells (Table 2). Therefore, SIRT2 had no major impact on immune cell development.

**Table 1:** Thymic cell subsets in SIRT2^+/+^ and SIRT2^−/−^ mice.

Cell subset	SIRT2^+/+^	SIRT2^−/−^
CD4^+^ CD8^+^	82.3 ± 3.1	82.9 ± 0.4
CD4^−^ CD8^−^	2.0 ± 0.6	1.8 ± 0.2
CD25^+^ CD44^+^	1.8 ± 0.6	1.6 ± 0.5
CD25^−^ CD44^+^	0.2 ± 0.01	0.2 ± 0.01
CD25^+^ CD44^−^	1.4 ± 0.6	1.3 ± 0.4
CD25^−^ CD44^−^	96.6 ± 1.2	96.9 ± 1.0
CD4^+^ CD8^−^	12.0 ± 2.3	12.1 ± 0.7
CD4^−^ CD8^+^	3.6 ± 0.3	3.3 ± 0.8

*Data are means ± SD of four animals per group expressed as the percentage of total cells (CD4^+^ CD8^+^, CD4^−^ CD8^−^, CD4^+^ CD8^−^, and CD4^−^ CD8^+^) or percentage of CD4^−^ CD8^−^ parental cells (CD25^+^ CD44^+^, CD25^−^ CD44^+^, CD25^+^ CD44^−^ and CD25^−^ CD44^−^). Total cell numbers were 49.2 ± 15.4 and 55.2 ± 5.7 millions per thymus in SIRT2^+/+^ and SIRT2^−/−^ mice, respectively. No statistically significant differences in subset percentages or absolute numbers were detected*.

**Table 2:** Splenic cell subsets in SIRT2^+/+^ and SIRT2^−/−^ mice.

Cell subset	SIRT2^+/+^	SIRT2^−/−^
CD3^+^ T cells (%)	27.3 ± 4.6	36.3 ± 4.8
CD4^+^	62.3 ± 2.7	61.5 ± 4.3
CD4^+^ CD44^low^ CD62L^high^ (naive)	46.0 ± 2.9	43.7 ± 6.8
CD4^+^ CD44^high^ CD62L^low^ (memory)	16.3 ± 2.9	17.8 ± 6.9
CD8^+^	31.5 ± 2.0	32.0 ± 2.9
CD8^+^ CD44^low^ CD62L^high^ (naive)	23.1 ± 0.5	23.5 ± 0.3
CD8^+^ CD44^high^ CD62L^low^ (memory)	8.4 ± 0.5	8.5 ± 0.3
CD4^−^ CD8^−^	1.3 ± 0.2	1.6 ± 0.2
B220^+^ B cells (%)	52.2 ± 7.4	51.3 ± 3.1
B220^+^ IgD^+^ CD23^+^ (mature)	45.6 ± 1.2	44.1 ± 0.9
B220^+^, non-IgD^+^/CD23^+^ (immature)	6.6 ± 1.2	7.2 ± 0.9
CD11c^+^ dendritic cells (%)	6.6 ± 0.2	6.4 ± 0.6
B220^−^	62.8 ± 2.5	62.8 ± 4.7
B220^+^	37.2 ± 2.5	37.2 ± 4.7
CD4^+^ CD25^+^ Foxp3^+^ Tregs (%)	4.5 ± 0.4	4.7 ± 0.2

*Data are means ± SD of four animals per group expressed as the percentage of CD3^+^, B220^+^, CD11c^+^, and CD4^+^ Foxp3^+^ splenic cells or the percentage of the CD3^+^, B220^+^, and CD11c^+^ parental populations expressing CD4, CD8, CD44, CD62L, IgD, and CD23. Total cell numbers were 74.2 ± 5.6 and 67.4 ± 8.7 millions per spleen in SIRT2^+/+^ and SIRT2^−/−^ mice, respectively. No statistically significant differences in subset percentages or absolute numbers were detected*.

**Figure 2:**
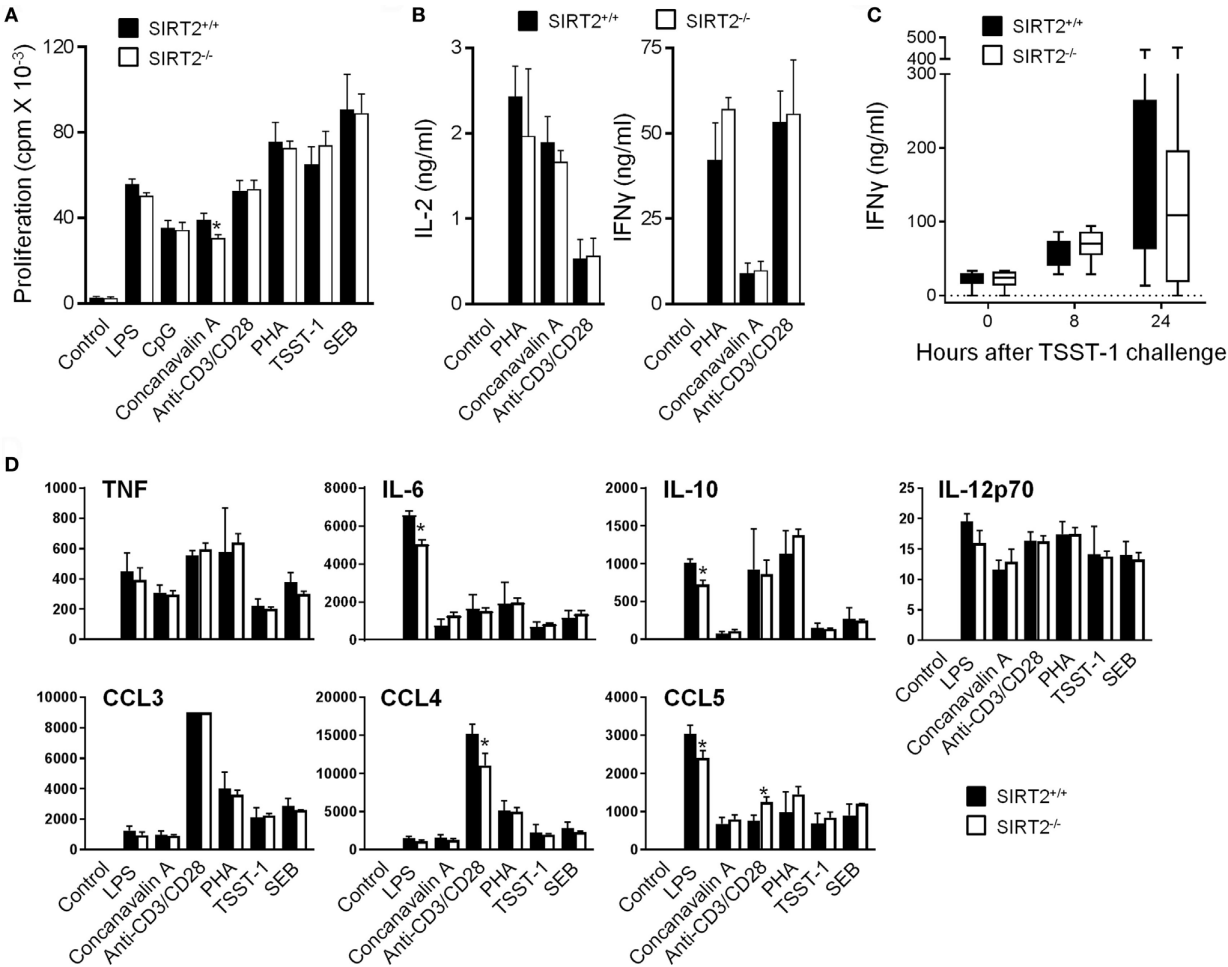
Sirtuin 2 (SIRT2) deficiency does not affect proliferation and cytokine response of splenocytes and IFNγ production in mice challenged with toxic shock syndrome toxin-1 (TSST-1). **(A,B)** SIRT2^+/+^ and SIRT2^−/−^ splenocytes were incubated for 48 h with lipopolysaccharide (LPS) (5 µg/ml), CpG (2 µg/ml), concanavalin A (5 µg/ml), anti-CD3/CD28 antibodies (1 µg/ml), phytohemagglutinin (PHA) (10 µg/ml), TSST-1 (2 µg/ml), and staphylococcal enterotoxin B (SEB) (5 µg/ml). Proliferation was measured by ^3^H-thymidine incorporation **(A)** while IL-2 and IFNγ concentrations in cell culture supernatants were quantified by ELISA **(B)**. Data are means ± SD of triplicate samples from one experiment performed with four mice and are representative of two experiments (*P < 0.05). **(C)** SIRT2^+/+^ and SIRT2^−/−^ mice (*n* = 8 per group) were injected with TSST-1 (0.5 mg/kg i.p.). Blood was collected after 0, 8, and 24 h to quantify IFNγ concentrations. Data are means ± SD. *P* > 0.5 for all time points. **(D)** SIRT2^+/+^ and SIRT2^−/−^ splenocytes were incubated for 48 h with LPS, CpG, concanavalin A, anti-CD3/CD28 antibodies, PHA, TSST-1, and SEB. TNF, IL-6, IL-10, IL-12p70, CCL3 (MIP-1α), CCL4 (MIP-1β), and CCL5 (RANTES) were quantified by Luminex. Data (in pg/ml) are means ± SD of one experiment performed with three mice. CCL3 values in response to anti-CD3/CD8 were over the upper limit of detection of the assay (*P < 0.05).

As a first approach to evaluate whether SIRT2 influenced immune responses, SIRT2^+/+^ and SIRT2^−/−^ splenocytes were exposed to microbial and immunological stimuli: LPS, CpG, concanavalin A, anti-CD3/CD28, PHA, TSST-1, and SEB. The proliferation and production of IL-2 and IFNγ (measured by ELISA) by splenocytes were not affected by SIRT2 deficiency (Figures 2A,B). In agreement, blood concentrations of IFNγ were similar in SIRT2^+/+^ and SIRT2^−/−^ mice injected with TSST-1 (Figure 2C), a staphylococcal superantigen responsible of toxic shock syndrome. A Luminex assay was then used to quantify TNF, IL-6, IL-10, IL-12p70, CCL3/Mip1α, CCL4/Mip1β, and CCL5/Rantes secretion by splenocytes exposed to LPS, CpG, concanavalin A, anti-CD3/CD28, PHA, TSST-1, and SEB (Figure 2D). No differences were observed between SIRT2^+/+^ and SIRT2^−/−^ splenocytes apart from a 20–28% reduction of LPS-induced IL-6, IL-10, and CCL5 and anti-CD3/CD28-induced CCL4, while the secretion of CCL5 was increased in response to anti-CD3/CD28.

### SIRT2 Deficiency Increases Phagocytosis by Macrophages

**Figure 3:**
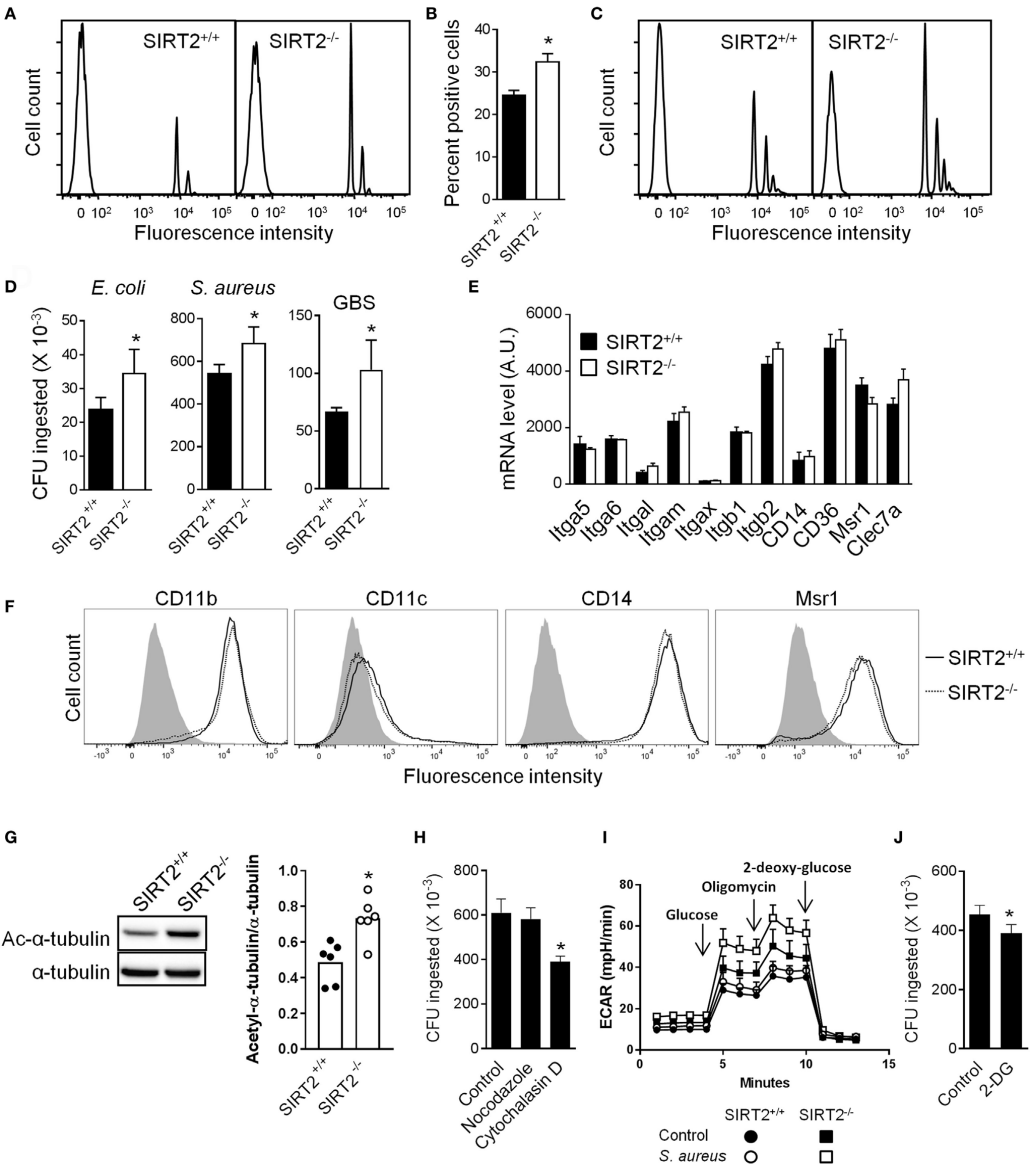
Sirtuin 2 (SIRT2) deficiency increases bacterial phagocytosis by macrophages. **(A–C)** SIRT2^+/+^ and SIRT2^−/−^ BM-derived macrophages (BMDMs) were incubated with 10 fluorescent beads [opsonized in **(C)**] per cell. After 1 h, cells were analyzed by flow cytometry. Representative histograms are depicted in **(A,C)**. The percentage of fluorescent cells was calculated **(B)**. Data are means ± SD from an experiment performed with four mice (*P = 0.002). **(D)** SIRT2^+/+^ and SIRT2^−/−^ BMDMs were incubated with live *Escherchia coli, Staphylococcus aureus*, and Group B *Streptococcus* (GBS) (10 bacteria/cell). Phagocytosis was assessed after 1 h. Data are means ± SD from one experiment performed with four (*E. coli* and GBS) or eight (*S. aureus*) mice. *P = 0.03, 0.006, and 0.03 for *E. coli, S. aureus*, and GBS, respectively. **(E)** Itga5, Itga6, Itgal, Itgam, Itgax, Itgb1, Itgb2, Cd14, Cd36, Msr1, and Clec7a mRNA expression levels in SIRT2^+/+^ and SIRT2^−/−^ BMDMs. Data are means ± SD of one experiment performed with three mice. **(F)** CD11b, CD11c, CD14, and Msr1 expression levels were analyzed by flow cytometry. The gray histogram represents staining with an isotype control antibody. **(G)** Expression levels of acetylated (Ac) and total tubulin in BMDMs were analyzed by western blotting and quantified by imaging. Data are means ± SD from six mice (right panel) (*P = 0.015). Full-length blots are presented in Figure S2 in Supplementary Material. **(H)** Phagocytosis of *S. aureus* [performed as in **(D)**] by SIRT2^−/−^ BMDMs preincubated for 1 h with or without nocodazole (10 µM) and cytochalasin D (10 µM). Data are means ± SD from one experiment performed with four mice (*P = 0.008). **(I)** Glycolytic capacity of BMDMs assessed by measuring the extracellular acidification rate (ECAR) using the Seahorse XF Glycolysis Stress Test Kit as described in Section “Materials and Methods.” Data are means ± SD from one experiment performed with three mice. **(J)** Phagocytosis of *S. aureus* [performed as in **(D)**] by SIRT2^−/−^ BMDMs preincubated for 1 h with or without 25 mM 2-deoxy-glucose (2-DG). Data are means ± SD from one experiment performed with four mice (*P = 0.017).

Macrophages are professional phagocytic cells that play a major role in antimicrobial host defenses. Therefore, we tested whether SIRT2 deficiency had an effect on phagocytosis by BMDMs. SIRT2^+/+^ and SIRT2^−/−^ BMDMs were incubated with fluorescent beads and analyzed by flow cytometry (Figures 3A–C). A higher percentage of SIRT2^−/−^ than SIRT2^+/+^ BMDMs phagocytosed beads (32.4 ± 1.9 vs 24.5 ± 1.2 percent positive cells, *P* = 0.002; Figures 3A,B), regardless of opsonization (Figure 3C). SIRT2^−/−^ BMDMs also exhibited higher phagocytosis using a panel of fluorescently labeled heat-inactivated bacteria (% of SIRT2^−/−^ vs SIRT2^+/+^ BMDMs ingesting bacteria: *E. coli* J5: 53.6 vs 43.6%, *E. coli* O111: 31.6 vs 23.0%, *Salmonella* Typhimurium: 24.1 vs 17.6%, *Neisseria meningitis*: 46.1 vs 37.2%, *S. pneumoniae*: 49.6 vs 34.7%). BMDMs were additionally exposed to live *E. coli, S. aureus* and GBS for 1 h before measuring the number of intracellular bacteria by plating cell lysates and enumerating colonies. Confirming the results obtained using inert beads and fluorescent bacteria, the numbers of phagocytosed *E. coli, S. aureus* and GBS were 1.3-fold to 1.6-fold higher using SIRT2^−/−^ BMDMs (Figure 3D).

Macrophages express phagocytic receptors, including macrophage scavenger receptor 1 (Msr1/SR-AI/CD204), CD14, CD36, C-type lectins such as dectin-1 (encoded by Clec7a), and members of the integrin superfamily (integrin α5/Itga5/CD49e, integrin αM/Itgam/CD11b, integrin αX/Itgax/CD11c, integrin β2/Itgb2/CD18). SIRT2^−/−^ and SIRT2^+/+^ BMDMs expressed comparable mRNA levels of Itga5, Itga6, Itgal, Itgam, Itgax, Itgb1, Itgb2, Cd14, Cd36, Msr1, and Clec7a (Figure 3E). Moreover, SIRT2^−/−^ and SIRT2^+/+^ BMDMs expressed similar levels of membrane-bound CD11b, CD11c, CD14, and Msr1 (Figure 3F). Hence, SIRT2 deficiency likely improved phagocytosis by BMDMs in a phagocytic receptor independent fashion.

Stabilization of microtubules and high glycolytic activity have been associated with efficient phagocytosis by macrophages (37–40). Since SIRT2 impacts on microtubules stabilization and glucose metabolism (8, 13, 41), we questioned whether these processes influenced phagocytosis by SIRT2^−/−^ BMDMs. In BMDMs, SIRT2 deficiency increased 1.5-fold tubulin acetylation (Figure 3G), a hallmark of microtubule stabilization. However, the microtubule destabilizer nocodazole did not impair the phagocytosis of *S. aureus* by BMDMs (Figure 3H), while the actin depolymerization agent cytochalasin D efficiently inhibited phagocytosis. Interestingly, the glycolytic activity was higher in SIRT2^−/−^ than SIRT2^+/+^ BMDMs exposed to *S. aureus* (Figure 3I). Moreover, 2-DG, which inhibits glycolysis, significantly reduced the phagocytosis of *S. aureus* by BMDMs (Figure 3J). Albeit preliminary, these results suggested that differences in the metabolic capacity of SIRT2^+/+^ and SIRT2^−/−^ BMDMs may provide a mechanism by which SIRT2 impedes phagocytosis.

### SIRT2 Deficiency Does Not Affect Cytokine Response of BMDMs Exposed to Microbial Ligands and Sensitivity of Mice to Endotoxemia

**Figure 4:**
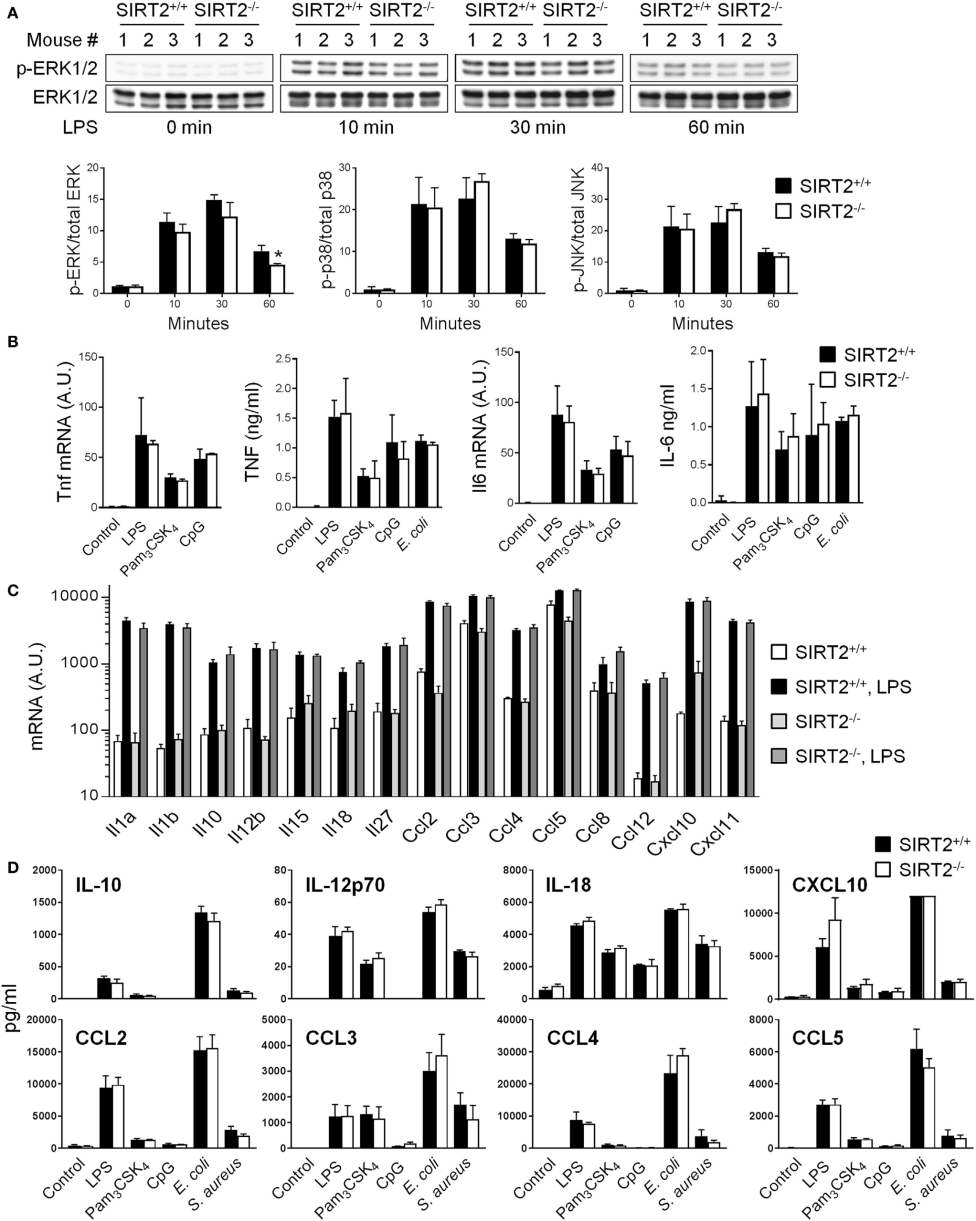
Sirtuin 2 (SIRT2) deficiency does not affect the response of macrophages to microbial stimulation. SIRT2^+/+^ and SIRT2^−/−^ BM-derived macrophages were exposed to lipopolysaccharide (LPS) (10 ng/ml), Pam_3_CSK_4_ (10 ng/ml), CpG (2 µg/ml), *Escherichia coli* (10^6^ CFU/ml), and *Staphylococcus aureus* (10^6^ CFU/ml). **(A)** Expression levels of phosphorylated (p) and total ERK1/2 (upper panel), p38 and JNK were analyzed by western blotting and quantified by imaging. Data are means ± SD from one experiment performed with three mice (lower panel) (*P = 0.02). **(B)** Tnf and Il6 mRNA levels and TNF and IL-6 concentrations in cell culture supernatants 1 and 8 h after stimulation, respectively. **(C)** Il1a, Il1b, Il10, Il12b, Il15, Il18, Il27, Ccl2/Mcp1, Ccl3/Mip1a, Ccl4/Mip1b, Ccl5/Rantes, Ccl8/Mcp2, Ccl12/Mcp5, Cxcl10/Ip10, Cxcl11/Itac mRNA levels after 8 h of culture with or without LPS. **(D)** IL-10, IL-12p70, IL-18, CXCL10, CCL2, CCL3, CCL4, and CCL5 concentrations in cell culture supernatants 8 h after stimulation measured by Luminex. Data are means ± SD of triplicate samples from one experiment performed with three mice (mRNA analyses) or six mice (TNF and IL-6 secretion), or means ± SD of single measurements from one experiment performed with three mice (Luminex). CXCL10 values in response to *E. coli* were over the upper limit of detection of the assay. No statistically significant differences were detected in **(B–D)**. A.U., arbitrary units. Full-length blots of panel **(A)** are presented in Figure S4 in Supplementary Material.

Sensing of microbial ligands through TLRs initiates MAPK and NF-κB signaling involved in the control of cytokine gene expression by innate immune cells (2). To address whether SIRT2 impacted intracellular signaling, the phosphorylation of ERK1/2, p38, and JNK MAPKs in BMDMs exposed to LPS for 0, 10, 30, and 60 min was analyzed by western blotting. The rate of LPS-induced phosphorylation of ERK1/2, p38 and JNK was very similar in SIRT2^+/+^ and SIRT2^−/−^ BMDMs, with only a slight and late reduction of ERK1/2 phosphorylation in SIRT2^−/−^ BMDMs (30% reduction at 1 hour) (Figure 4A). The nuclear translocation of phosphorylated NF-κB p65 was not different in SIRT2^+/+^ and SIRT2^−/−^ BMDMs exposed to LPS for 0, 10, 30, and 60 min (*P* > 0.5 for all time points).

SIRT2^+/+^ and SIRT2^−/−^ BMDMs exposed to LPS, Pam_3_CSK_4_, CpG (i.e., TLR4, TLR1/2 and TLR9 ligands, respectively), and *E. coli* upregulated Tnf and Il6 mRNA levels and secreted TNF and IL-6 to the same extend (Figure 4B). Additionally, Tlr1, Tlr2, Tlr4, and Tlr9 mRNA were modulated likewise in SIRT2^+/+^ and SIRT2^−/−^ BMDMs (Figure S3 in Supplementary Material). SIRT2^+/+^ and SIRT2^−/−^ BMDMs expressed also comparable mRNA levels of Il1a, Il1b, Il10, Il12b, Il15, Il18, Il27, Ccl2/Mcp1, Ccl3/Mip1a, Ccl4/Mip1b, Ccl5/Rantes, Ccl8/Mcp2, Ccl12/Mcp5, Cxcl10/Ip10, and Cxcl11/Itac at baseline and following LPS stimulation (Figure 4C), and secreted comparable levels of IL-10, IL-12p70, IL-18, CXCL10, CCL2, CCL3, CCL4, and CCL5 upon exposure to LPS, Pam_3_CSK_4_, CpG, *E. coli*, and *S. aureus* (Figure 4D). Altogether, these results argued against an important role of SIRT2 in controlling proinflammatory and anti-inflammatory cytokine response by macrophages exposed to TLR ligands.

**Figure 5:**
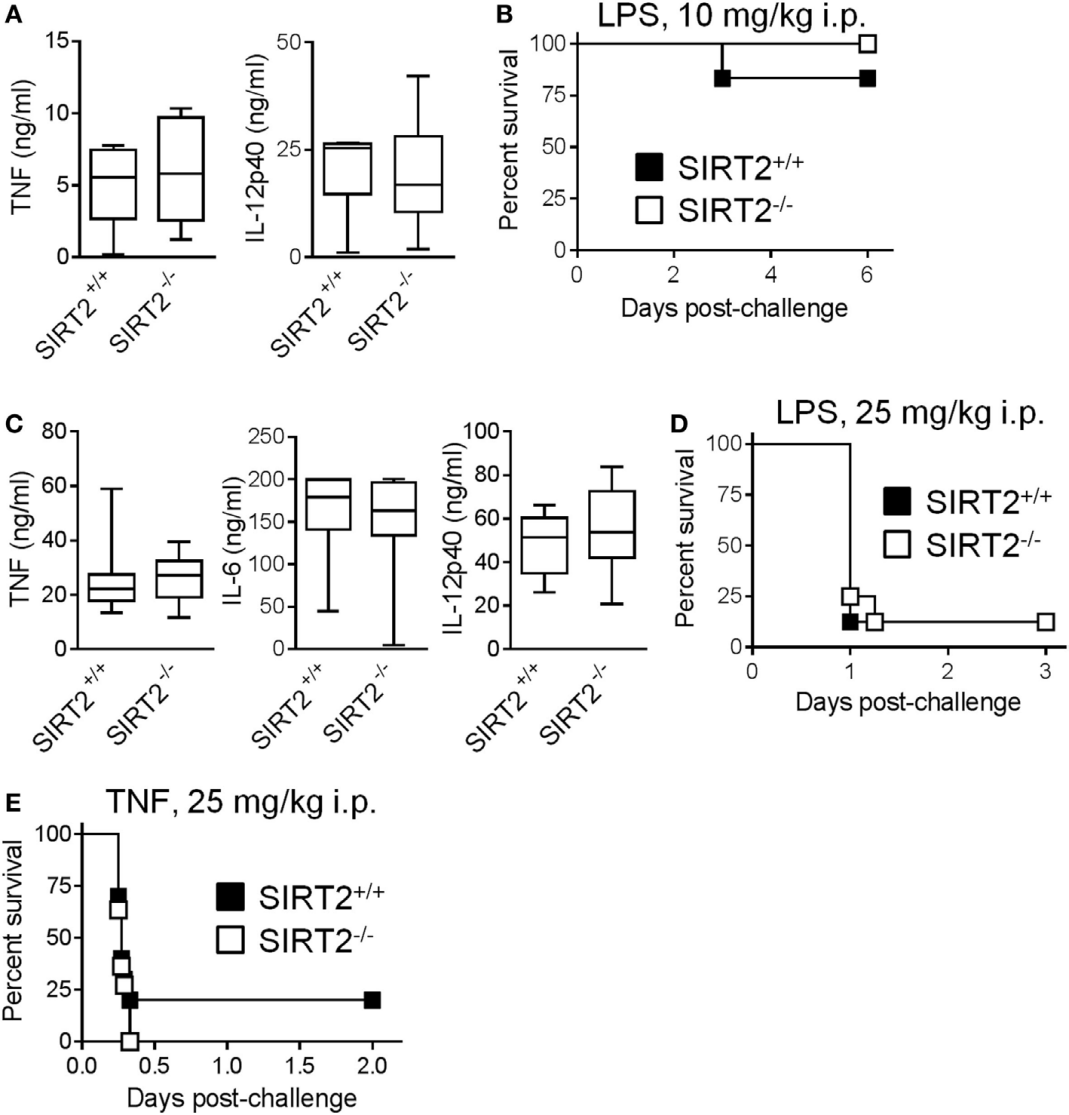
Sirtuin 2 (SIRT2) deficiency does not affect endotoxemia and TNF-induced shock. SIRT2^+/+^ and SIRT2^−/−^ mice were injected i.p. with 10 mg/kg lipopolysaccharide (LPS) [**(A,B)**, *n* = 6 per group] and 25 mg/kg LPS (**C,D**, *n* = 8 per group). **(A,C)** TNF, IL-6, and IL-12p40 concentrations in blood collected 1 h (TNF) and 6 h (IL-6 and IL-12p40) after LPS challenge. *P* > 0.5 for all conditions. **(B,D)** Survival of mice. *P* = 0.3 and 0.9. **(E)** Survival of SIRT2^+/+^ and SIRT2^−/−^ mice (*n* = 11 per group) sensitized with 30 mg/kg d-galactosamine and challenged i.p. with 25 mg/kg TNF. *P* = 0.6.

To assess the relevance of these observations *in vivo*, we developed models of endotoxemia of different severity (Figures 5A–D). In a mild model of endotoxemia (induced by an i.p. challenge with 10 mg/kg LPS), TNF and IL-12p40 concentrations in blood and mortality rates (83 vs 100%, *P* = 0.3) were comparable in SIRT2^+/+^ and SIRT2^−/−^ mice (Figures 5A,B). In a severe model of endotoxemia (induced by 25 mg/kg LPS), TNF, IL-6, and IL-12p40 concentrations in blood and mortality rates (88% in both groups, *P* = 0.69) were strongly increased, but remained similar in SIRT2^+/+^ and SIRT2^−/−^ mice (Figures 5C,D). Furthermore, SIRT2^+/+^ and SIRT2^−/−^ mice were equally sensitive to fulminant shock induced by TNF (25 mg/kg i.p. in d-galactosamine sensitized mice), the main driver of the lethal effect of endotoxemia (*P* = 0.6; Figure 5E). Overall, SIRT2 did not interfere with endotoxemia.

### SIRT2 Deficiency Protects from Chronic Staphylococcal Infection

Considering that SIRT2 impacted phagocytosis but not cytokine expression, we hypothesized that SIRT2 deficiency should provide some benefit during chronic lethal infection but not fulminant sepsis, and should not sensitize to benign infection. Therefore, we compared the impact of SIRT2 deficiency during rapidly lethal, sub-lethal, and chronic bacterial infections induced by *E. coli, K. pneumonia*, and *S. aureus*, three of the most frequent causes of bacterial sepsis in humans (42).

**Figure 6:**
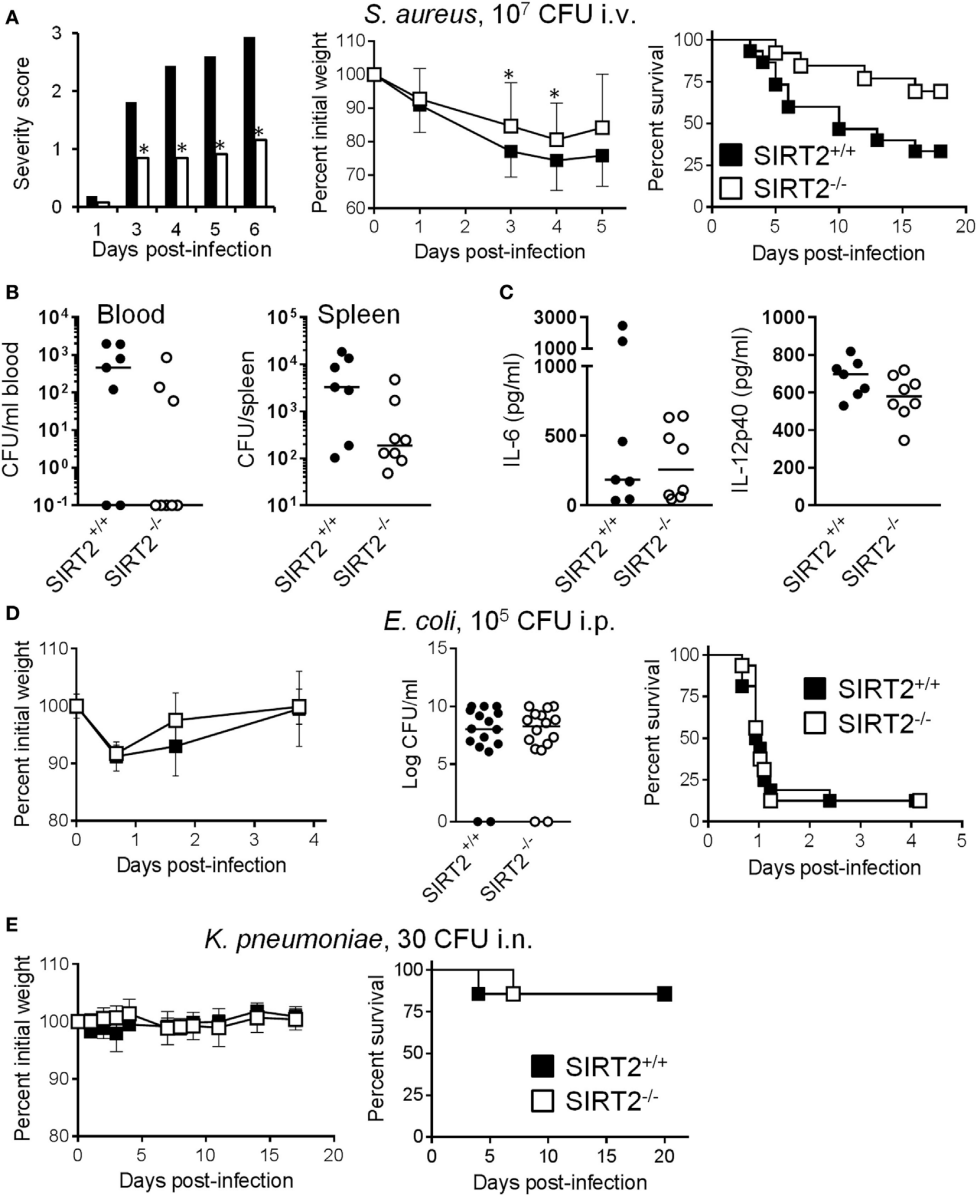
SIRT2 deficiency protects from chronic *Staphytococcus aureus* infection. **(A)** Severity score (*P ≤ 0.01), body weight (*P = 0.03 and 0.04) and survival (*P* = 0.04) of SIRT2^+/+^ and SIRT2^−/−^ mice challenged i.v. with 10^7^ CFU *S. aureus* (*n* = 13 and 9). **(B,C)** Blood and spleen were collected from SIRT2^+/+^ and SIRT2^−/−^ mice (*n* = 7 and 8) 48 h after *S. aureus* challenge. Bacteria were quantified in blood and spleen **(B)** while IL-6 and IL-12p40 concentrations were quantified in blood **(C)**. Horizontal bars represent the medians. *P* = 0.07 and 0.04 in **(B)**, and *P* = 0.95 and 0.12 in **(C)**. **(D)** Body weight, bacteria in blood 18 h postinfection (*P* = 0.9), and survival (*P* = 0.8) of SIRT2^+/+^ and SIRT2^−/−^ mice challenged i.p. with 10^5^ CFU *E. coli* (*n* = 16 per group). **(E)** Body weight and survival (*P* = 0.9) of SIRT2^+/+^ and SIRT2^−/−^ mice challenged i.n. with 30 CFU *K. pneumoniae* (*n* = 7 per group).

Supporting our working hypothesis, in a model of chronic infection by *S. aureus* in which mortality occurred 3 to 16 days post i.v. challenge with the bacteria, severity score, body weight loss, and survival (SIRT2^+/+^ vs SIRT2^−/−^: 33.3 vs 69.2%; *P* = 0.04) were all markedly improved in SIRT2^−/−^ mice (Figure 6A). In agreement with these findings, 48 h postinfection, only 37.5% (3/8) of SIRT2^−/−^ mice were bacteremic while 71.4% (5/7) of SIRT2^+/+^ mice were bacteremic (Figure 6B). Moreover, bacterial burden in the spleen was much lower in SIRT2^−/−^ than in SIRT2^+/+^ mice (1.9 × 10^2^ vs 3.3 × 10^3^ mean CFU/organ; *P* = 0.04). TNF was not detected in blood, while IL-6 and IL-12p40 levels were not different between SIRT2^−/−^ and SIRT2^+/+^ mice, although there was a trend toward lower IL-12p40 levels in SIRT2^−/−^ mice (Figure 6C). In a model of fulminant, rapidly lethal peritonitis induced by *E. coli*, body weight loss, bacterial dissemination into the blood, and survival rate (12.5% in both groups, *P* = 0.7) were comparable in SIRT2^+/+^ and SIRT2^−/−^ mice (Figure 6D). In a non-severe model of *K. pneumoniae* pneumonia, body weight loss and survival (85.7% in both groups, *P* = 0.9) were not affected by SIRT2 deficiency (Figure 6E). Finally, we questioned whether SIRT2 influenced host susceptibility to a non-bacterial chronic infection. Candidiasis was induced by i.v. inoculation of 10^5^ CFU/ml *C. albicans* into SIRT2^+/+^ and SIRT2^−/−^ mice (*n* = 14 and 16). Mice died 9–40 days postinfection, without survival differences between the SIRT2^+/+^ and SIRT2^−/−^ groups (71 vs 56%; *P* = 0.4), suggesting that SIRT2 deficiency did not compromise host defenses to *Candida* infection.

## Discussion

In the present study, we identified a unique role for SIRT2 in host–pathogen interactions. SIRT2 deficiency promoted bacterial phagocytosis by macrophages but not cytokine production. In agreement with these findings, SIRT2 deficiency protected from chronic staphylococcal infection while having no influence on the course of endotoxemia, TNF-induced shock, fulminant bacterial peritonitis, non-severe bacterial pneumonia, and chronic candidiasis. These observations are particularly relevant in light of the development of pharmacological inhibitors of SIRT2 for clinical applications (43), as they suggest that their usage would not increase susceptibility to bacterial and candidal infections.

SIRT2 was the most highly expressed sirtuin in myeloid cells. Macrophages expressed elevated levels of SIRT2, in accordance previous reports describing SIRT2 expression in microglial cells *in vivo* (16, 17). Interestingly, SIRT2 deficiency had no major impact on LPS-induced MAPK activation, NF-κB nuclear translocation, and cytokine production in macrophages. Moreover, cytokine levels in blood were similar in SIRT2^+/+^ and SIRT2^−/−^ endotoxemic mice. Likewise, inflammatory parameters were comparable in SIRT2^+/+^ and SIRT2^−/−^ mice with experimental stroke and *Mycobacterium tuberculosis* infection (21, 44). However, contradictory findings have been reported in the literature. While SIRT2 deficiency promoted NF-κB p65 acetylation and p65-dependent gene expression, it was also reported to reduce NF-κB and p38 and JNK MAPKs activation through an increased stability of IκB and activity of MAPK phosphatase-1, respectively (14–16, 18, 20, 45). In these studies, SIRT2 deficiency sustained brain inflammation, colitis, and collagen-induced arthritis, but protected from renal and liver inflammation (15, 16, 18, 20, 45).

The discrepancy of the effects of SIRT2 on inflammatory responses mirrors conflicting results observed for other sirtuins. For example, SIRT1 protected from experimental autoimmune encephalomyelitis, arthritis, lung inflammation, hepatic steatosis, and insulin resistance, but promoted lupus, arthritis, allergic airway disease, and allograft rejection (46–54). SIRT6 has not only been reported to protect against liver fibrosis, atherosclerosis, osteoarthritis, and arthritis but has also been associated with increased TNF production and the development of autoimmune encephalomyelitis and cerebral ischemia (55–62). Different experimental conditions might explain these apparent conflicting results, for example qualitative and quantitative differences in caloric input or subtle variations of NAD^+^ availability influencing sirtuin activity indirectly. Moreover, SIRT1, SIRT3, and SIRT6 modulate circadian function and are affected by circadian oscillation in the abundance of NAD^+^ (63, 64). Furthermore, sirtuins might primarily play a role in the development of long-lasting, chronic metabolic, and/or inflammation-related disorders while having a modest impact during acute infectious processes. For instance, SIRT3 deficiency has been reported to increase insulin resistance, diabetic cardiac dysfunction, allograft graft injury, and lung fibrosis, but had no impact on innate immune responses and susceptibility to endotoxemia or bacterial and fungal sepsis (65–73).

Two prior studies examined SIRT2 in the context of infection by intracellular bacteria. SIRT2 deletion in the myeloid compartment had no noticeable impact on host defenses against *M. tuberculosis* infection as attested by cellular infiltrates, cytokine expression, and long-term bacterial burden in lungs (44). *Listeria monocytogenes* promoted SIRT2-dependent histone H3 deacetylation and redirected host gene expression to favor infection (74). Whether other microorganisms subvert SIRT2 or other sirtuins at their own benefit is unknown.

We analyzed host responses to extracellular bacteria most frequently isolated from septic patients. Strikingly, SIRT2 deficiency enhanced the engulfment of Gram-positive and Gram-negative bacteria by macrophages, an effect apparently unrelated with a differential expression of phagocytic receptors or microtubule polymerization. This observation was surprising considering that on the one hand SIRT2 deacetylates α-tubulin and destabilizes the microtubule network (8, 41) and on the other end microtubule depolymerizating agents were reported to inhibit phagocytosis (75, 76). Yet, the effects of microtubule depolymerizating agents were tested using an immortalized macrophage-like mouse cell line and human neutrophils that may behave differently than primary BMDMs. As a positive control (77), actin depolymerization efficiently inhibited phagocytosis by BMDMs. Increased glycolysis has been associated with efficient phagocytosis by macrophages (39, 40). SIRT2 deficiency reduced HIF-1α deacetylation and destablization (78), and augmented glycolysis in human fibroblasts (79). We observed that SIRT2 deficiency increased glycolysis and that glycolysis inhibition reduced phagocytosis in BMBMs. Albeit preliminary, these results suggest that SIRT2 may influence phagocytosis through metabolic constraints.

The improved control of bacterial burden during chronic staphylococcal infection might be related to improved phagocytosis but also to enhanced autophagy in SIRT2^−/−^ mice. Autophagy facilitates the clearance of cytoplasmic bacteria (80) and has been involved in host defenses and tolerance to *S. aureus* infection (81, 82). Hyperacetylation of tubulin stimulated autophagy upon nutrient deprivation, and SIRT2 deficiency increased autophagy in a colorectal cancer cell line (83, 84). Thus, by regulating tubulin acetylation and metabolic activity, SIRT2 may contribute to modulate phagocytic and autophagy defense mechanisms, though the latter has not been formally demonstrated.

From a translational perspective, it was important to define the impact of SIRT2 in preclinical models of infection. A main observation of this study is that SIRT2 deficiency protected mice from chronic staphylococcal infection, while it neither protected nor sensitized mice to TNF-induced shock, endotoxemia, rapidly lethal *E. coli* peritonitis and mild *K. pneumoniae* pneumonia. Additionally, SIRT2 deficiency did not influence the development of chronic candidiasis as it did for chronic staphylococcal infection. This may not be surprising considering differences in host–pathogen interactions during fungal and bacterial infections. Whether SIRT2 protects from other chronic bacterial infections should be tested in the future. Nonetheless, these results support the clinical development of SIRT2 inhibitors regarding their infection-related safety profile. This contrasts with inhibitors of HDAC1-11 that impaired innate immune defenses, increased susceptibility to infection in preclinical mouse models and have been associated with severe infections in patients (29, 85–89).

Overall, SIRT2 has a subtle impact on host defense responses to bacterial infections. Considering that sirtuins are intricately linked with metabolism, age-associated dysfunctions and lifespan, it will be important to analyze the impact of SIRT2 on host defenses under metabolic stress conditions and according to age. To conclude, our results are encouraging with respect to developing inhibitors of SIRT2, which are safe in terms of susceptibility to infections, for treating metabolic and neurodegenerative diseases, such as Parkinson’s disease and Huntington’s disease (90, 91).

## Ethics Statement

Animal experimentation was approved by the Service de la Consommation et des Affaires Vétérinaires (SCAV) du Canton de Vaud (Epalinges, Switzerland) under authorizations no. 876.8 and 877.8, and performed according to Swiss and ARRIVE guidelines.

## Author Contributions

EC, TH, CT, JH, MM, JL, MP, BT, and SL performed *in vitro* experiments. EC, TH, CT, and DLR performed *in vivo* experiments. HA-O and JA contributed to reagents. TR conceived the project, designed the experiments, and wrote the paper. All the authors revised the paper.

## Conflict of Interest Statement

The authors declare that the research was conducted in the absence of any commercial or financial relationships that could be construed as a potential conflict of interest.
