# Supplementary material for: Sirtuin 2 Deficiency Increases Bacterial Phagocytosis by Macrophages and Protects from Chronic Staphylococcal Infection
Source: Front Immunol. 2017 Aug 28;8:1037. doi: 10.3389/fimmu.2017.01037 (PMC5581327; doi:10.3389/fimmu.2017.01037)

Supplementary Figure S1

Full-length blots

Panels used in Figure 1E

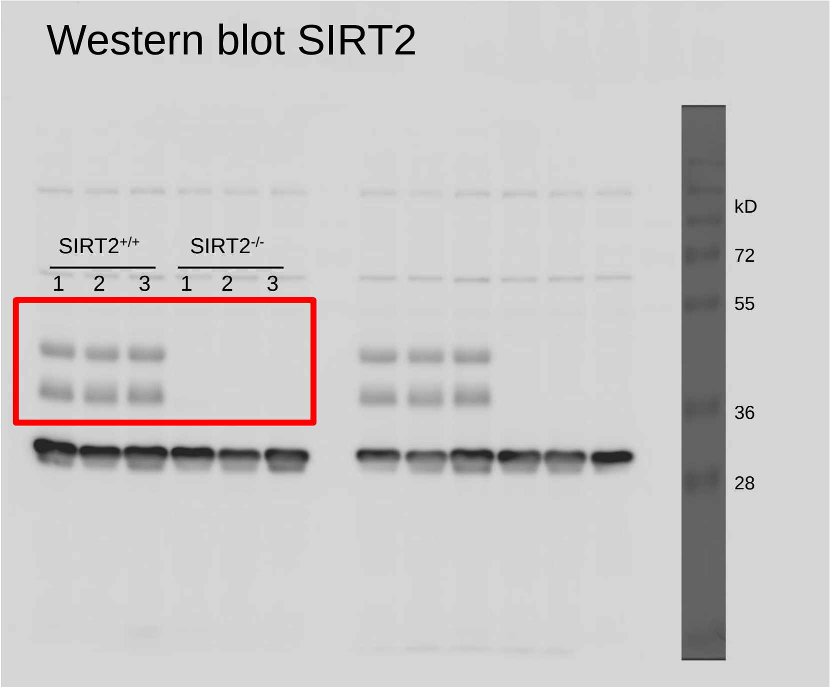

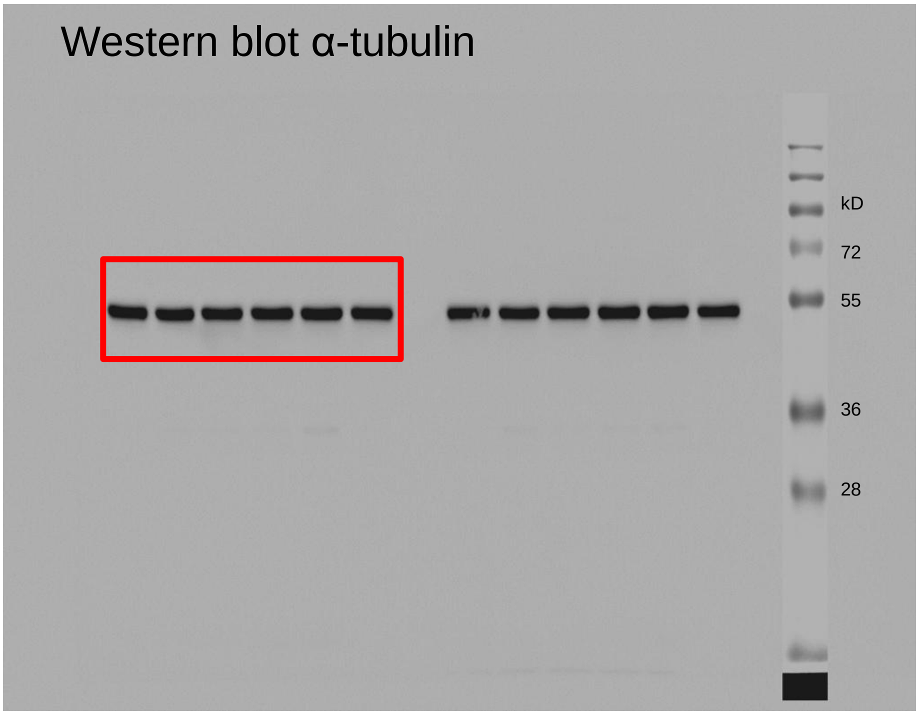

Supplementary Figure S2

Full-length blots

Panels used in Figure 3G

Western blot acetylated α-tubulin

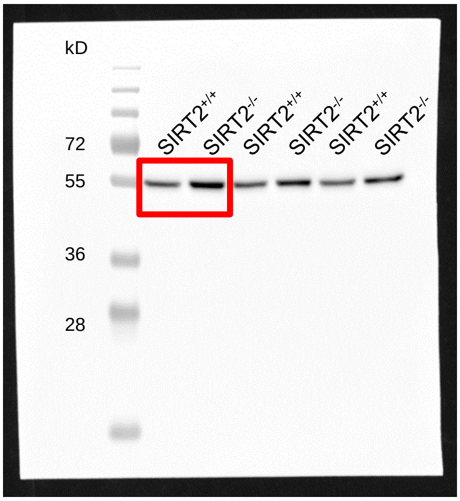

Western blot α-tubulin

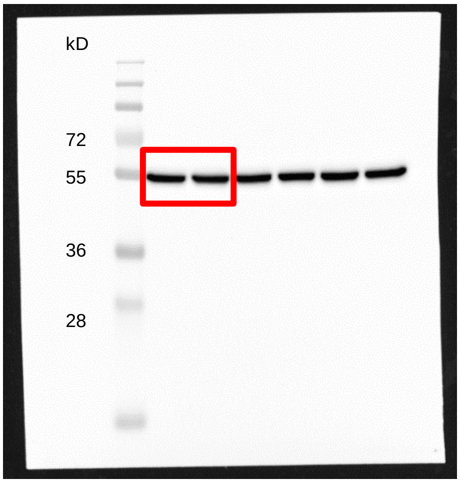

Supplementary Figure S3

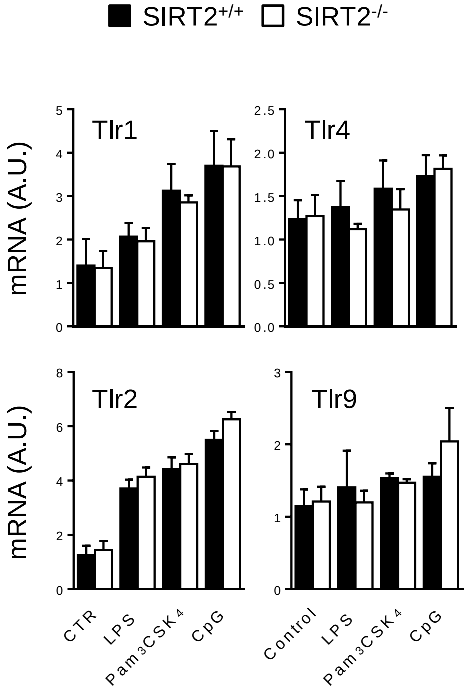

SIRT2 deficiency does not affect the expression of TLRs by macrophages to microbial stimulation. SIRT2$^{+/+}$ and SIRT2$^{-/-}$ BMDMs were exposed for 1 hour to LPS (10 ng/ml), Pam_3CSK_4 (10 ng/ml) and CpG (2 µg/ml). Tlr1, Tlr2, Tlr4 and Tlr9 mRNA levels were quantified by real-time PCR. Data are means ± SD of triplicate samples from one experiment performed with 3 mice.

Supplementary Figure S4

Full-length blots

Panels used in Figure 4A

Western blots
phospho-ERK1/2

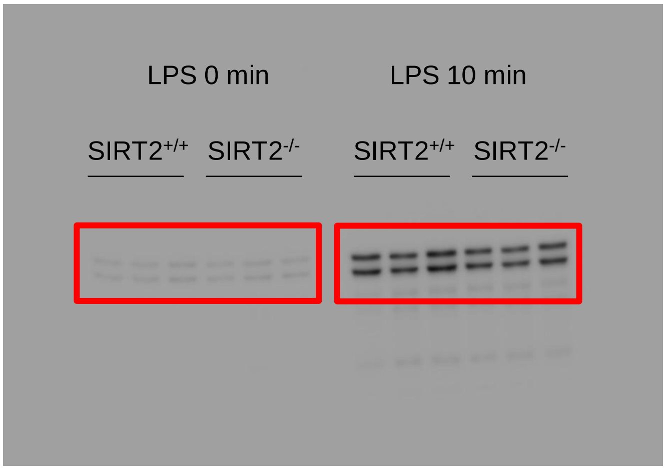

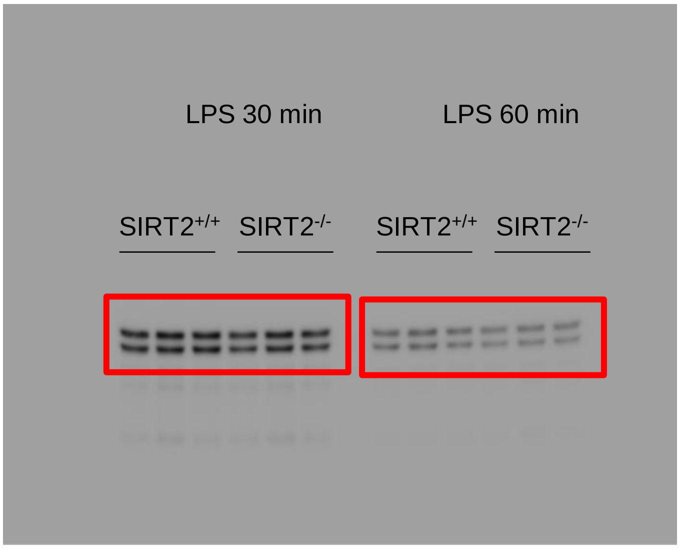

Western blots
total ERK1/2

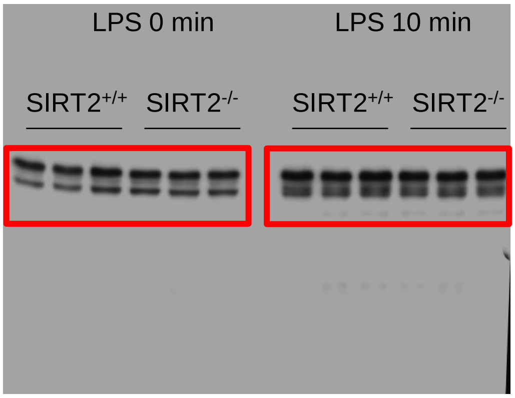

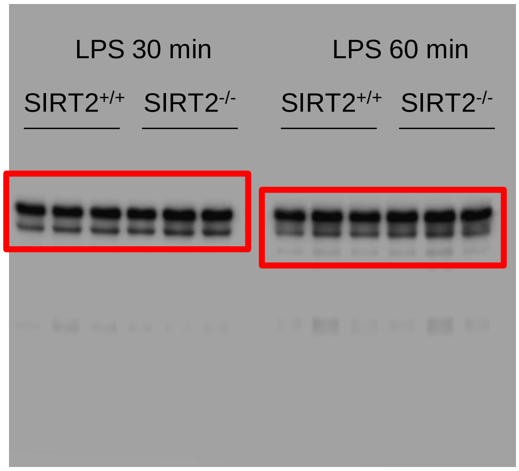

Supplement: Supplementary file 1 [file Presentation_1.PDF]
