# Supplementary material for: Sirtuin 2 Deficiency Increases Bacterial Phagocytosis by Macrophages and Protects from Chronic Staphylococcal Infection
Source: Front Immunol. 2017 Aug 28;8:1037. doi: 10.3389/fimmu.2017.01037 (PMC5581327; doi:10.3389/fimmu.2017.01037)
Supplement: Supplementary file 2 [file Data_Sheet_1.docx]

**Supplementary Table S1. Oligonucleotides used to quantify Sirt1-7 mRNA levels by RT-PCR**

| Target | Forward (5’-3’) | Reverse (5’-3’) |
| --- | --- | --- |
| Sirt1 | **agggaacctttgcctcatct** | **gaggtgttggtggcaactct** |
| Sirt2 | **gaggtggcatggattttgac** | **agatggtagtgctggggttg** |
| Sirt3 | **acagctacatgcacggtctg** | **gggaggtcccaagaatgagt** |
| Sirt4 | **cgagcaaaagctcccaatag** | **gatcttgagcagcggaactc** |
| Sirt5 | **ggccgagtttaacatggaga** | **ccgttagtgccctgctttag** |
| Sirt6 | **acctgcaacccacaaaacat** | **ggctcagccttgagtgctac** |
| Sirt7 | **cacatgagcatcacccgttt** | **agcccatcacagttctgagaca** |

**Supplementary Table S2. Antibodies used for flow cytometry analyses**

| Purpose | Target | Clone name | Coupling/reference |
| --- | --- | --- | --- |
| Flow cytometry | B220 | RA3-6B2 | eFluor® 450 |
|  | CD3 | 145-2C11 | PE, eFluor® 450 |
|  | CD4 | RM4-5 | PE, FITC |
|  | CD8 | 53-6.7 | APC-eFluor® 780, APC-Cy7 |
|  | CD11b | M1/710 | PE, APC |
|  | CD11c | HL3 | PE, APC |
|  | CD14 | Sa2-8 | PE-Cy7 |
|  | CD23 | B3B4 | PE |
|  | CD25 | PC61.5 | APC |
|  | CD36 | 72-1 | PE |
|  | CD44 | IM7 | APC, eFluor® 450 |
|  | CD62L | MEL-14 | FITC |
|  | CD93 | AA4.1 | APC |
|  | CD204 | 2F8 | FITC |
|  | IgD | AMS 9.1 | FITC |
|  | MHC-II | 114.15.2 | FITC |
|  |  |  |  |
| Western blot | α-tubulin | B-5-1-2 | T5168 |
|  | Acetylated α-tubulin | 6-11B-1 | sc-23950 |
|  | ERK1/2 | polyclonal | 9102 |
|  | JNK | polyclonal | 9252 |
|  | NF-κBp65 | D14E12 | 8242 |
|  | Phospho-NF-κBp65 | 93H1 | 3033 |
|  | p38 | polyclonal | 9212 |
|  | Phospho-ERK1/2 | polyclonal | 9101 |
|  | Phospho-JNK | polyclonal | 9251 |
|  | Phospho-p38 | polyclonal | 9211 |
|  | SIRT2 | polyclonal | ab67299 |
|  | TBP | polyclonal | 8515 |

PE: phycoerythrin; FITC: fluorescein isothiocyanate; APC: allophycocyanin. Antibodies for flow cytometry were from eBioscience, except the anti-CD204 and anti-IgD, which were from Bio-Rad and BD Biosciences (Pharmingen), respectively. Antibodies for Western blot were from Cell Signaling Technology, except the anti-α tubulin, anti-acetylated α tubulin and anti-SIRT2, which were from Sigma-Aldrich, Santa Cruz Biotechnology (Dallas, TX) and Abcam, (Cambridge, UK), respectively.
